# *De Novo* Assembly and Characterization of Stress Transcriptome in a Salinity-Tolerant Variety CS52 of *Brassica juncea*

**DOI:** 10.1371/journal.pone.0126783

**Published:** 2015-05-13

**Authors:** Rita Sharma, Manjari Mishra, Brijesh Gupta, Chirag Parsania, Sneh L. Singla-Pareek, Ashwani Pareek

**Affiliations:** 1 Stress Physiology and Molecular Biology Laboratory, School of Life Sciences, Jawaharlal Nehru University, New Delhi, India; 2 Plant Molecular Biology Group, International Centre for Genetic Engineering and Biotechnology, Aruna Asaf Ali Marg, New Delhi, India; 3 Bionivid Technology [P] Ltd., Bangalore, India; University of Delhi South Campus, INDIA

## Abstract

Oilseed mustard, *Brassica juncea*, exhibits high levels of genetic variability for salinity tolerance. To obtain the global view of transcriptome and investigate the molecular basis of salinity tolerance in a salt-tolerant variety CS52 of *B*. *juncea*, we performed transcriptome sequencing of control and salt-stressed seedlings. *De novo* assembly of 184 million high-quality paired-end reads yielded 42,327 unique transcripts longer than 300 bp with RPKM ≥1. When compared with non-redundant proteins, we could annotate 67% unigenes obtained in our study. Based on the mapping to expressed sequence tags (ESTs), 52.6% unigenes are novel compared to EST data available for *B*. *juncea* and constituent genomes. Differential expression analysis revealed altered expression of 1469 unigenes in response to salinity stress. Of these, 587, mainly associated with ROS detoxification, sulfur assimilation and calcium signaling pathways, are up regulated. Notable of these is *RSA1 (SHORT ROOT IN SALT MEDIUM 1) INTERACTING TRANSCRIPTION FACTOR 1 *(*RITF1*) homolog up regulated by >100 folds in response to stress. *RITF1*, encoding a bHLH transcription factor, is a positive regulator of *SOS1* and several key genes involved in scavenging of salt stress-induced reactive oxygen species (ROS). Further, we performed comparative expression profiling of key genes implicated in ion homeostasis and sequestration (*SOS1*, *SOS2*, *SOS3*, *ENH1*, *NHX1*), calcium sensing pathway (*RITF1*) and ROS detoxification in contrasting cultivars for salinity tolerance, *B*. *juncea* and *B*. *nigra*. The results revealed higher transcript accumulation of most of these genes in *B*. *juncea* var. CS52 compared to salt-sensitive cultivar even under normal growth conditions. Together, these findings reveal key pathways and signaling components that contribute to salinity tolerance in *B*. *juncea *var. CS52.

## Introduction

*Brassica juncea* (L.) Czern and Coss, also known as Indian mustard, is mainly cultivated as an oilseed crop. Like other crop plants grown in the arid and semiarid regions of the world, yield and product quality in *Brassica* species is adversely affected by a wide range of biotic and abiotic stresses [1,2]. Singh and coworkers [3] reported significant reductions in oil, protein and fiber contents with increased erucic acid content in *Brassica* cultivars in response to salt stress. Therefore, identification and generation of improved genotypes with increased salinity tolerance is required to maintain the optimum yield and product quality in *Brassica* species.

*B*. *juncea* exhibits significant intraspecific genetic variability for salinity tolerance [4,5]. CS52, an Indian mustard variety developed at Central Soil Salinity Research Institute (CSSRI; http://cssri.nic.in/achievements.htm) India, is considerably adapted to saline and sodic soils (EC_e_ 6.0–8.5 dS m^-1^ and sodicity pH_2_9.3). Physiological experiments revealed the maximum accumulation of proline with minimum levels of H_2_O_2_, electrolyte leakage and malondialdehyde contents in CS52 compared to other varieties tested [6]. Based on the expression analysis of a limited number of genes, it has been earlier proposed that SOS2 pathway is likely responsible for salinity tolerance in this variety [7,8]. However, salinity tolerance is a very complex trait regulated by several independent and/or interdependent pathways. Therefore, whole genome expression profiling is needed to investigate the global changes in gene expression in response to salinity stress and understand the functional genetic basis of salinity tolerance in *B*. *juncea* var. CS52.

*B*. *juncea* is a natural amphidiploid (AABB, 2n = 36) of *B*. *rapa* (AA, 2n = 20) and *B*. *nigra* (BB, 2n = 16) with haploid (1X) genome size estimated to be 534 Mbp [9]. So far, the whole genome sequence of only *B*. *rapa* has been attempted [10] with very limited sequence information available for *B*. *juncea* and *B*. *nigra* genomes in public databases. Given the large genome size, limited sequence information and genomic resources available, exploiting genetic potential for genome-assisted breeding and trait improvement in *B*. *juncea* is greatly hampered. As a significant step towards deciphering global view of transcriptome and comparative expression analysis in *B*. *juncea*, recently RNA sequencing followed by *de novo* assembly has been adopted. Sun and coworkers [11] used Illumina short-read technology with a tag-based digital gene expression system to gain insights into the molecular mechanism of stem swelling and development in the tumorous stem mustard *B*. *juncea* var. tumida Tsen et Lee. Similarly, Liu and coworkers [12] employed RNA sequencing to investigate the molecular mechanism underlying seed pigmentation in *B*. *juncea*. Recently, Paritosh and coworkers [13] developed SNP markers for Varuna and Heera lines of *B*. *juncea* and analyzed synteny between two constituent genomes using RNA-sequencing data. However, so far, genome-wide analysis of gene expression in response to abiotic stresses has not been undertaken in *B*. *juncea*.

Here, we report transcriptome sequencing of two-weeks-old seedlings of *B*. *juncea* var. CS52 under normal growth conditions (CTRL) and in response to salinity stress (SS). Analysis of high expressing genes in CTRL library and differentially expressed genes in response to salinity stress revealed key signaling components and pathways contributing to salinity tolerance. Real-time qPCR-based expression analysis of key abiotic stress-responsive genes in two contrasting cultivars of *Brassica* (salt-tolerant, *B*. *juncea* and salt-sensitive, *B*. *nigra*) highlighted constitutive expression of most of them in the tolerant variety under normal growth conditions. Furthermore, data generated in this study provides a valuable resource for more focused investigations of salinity tolerance mechanisms in *Brassica* genotypes and initiating functional genomic studies for trait improvement.

## Materials and Methods

### Plant materials and stress treatment

Seeds of *B*. *juncea* var. CS52 were washed with de-ionized water and germinated in a hydroponic system with continuous air bubbling for 48 h in dark and then transferred to light for further growth (25 ± 2°C, 12 h light and dark cycle) in plant growth room. Fifteen-day-old seedlings were subjected to 200 mM salinity stress for 24 h. After 24 h, tissue from about ten randomly selected seedlings was pooled to minimize biological variability for each sample and frozen in liquid nitrogen. Seedlings maintained in water were simultaneously collected as control.

### RNA extraction, library preparation and sequencing

Total RNA was extracted from the frozen tissue using TRIzol reagent (Sigma, USA) as per manufacturer’s instructions. Extracted RNA was assessed for quality and quantity using an Agilent 2100 Bioanalyzer (Agilent Technologies). RNA with an RNA Integrity Number (RIN) ≥8.0 was used for mRNA purification using oligo dT beads (TruSeq RNA Sample Preparation Kit, Illumina). The purified mRNA was fragmented at elevated temperature (90°C) in the presence of divalent cations and reverse transcribed with Superscript II Reverse Transcriptase (Invitrogen Life Technologies) by priming with random hexamers. Second strand cDNA was synthesized in the presence of DNA polymerase I and RNaseH. The cDNA was cleaned using Agencourt Ampure XP SPRI beads (Beckman Coulter). Illumina adapters were ligated to the cDNA molecules after end repair and addition of an ‘A’ base followed by SPRI clean-up. The resultant cDNA library was amplified using PCR for enrichment of adapter ligated fragments, quantified using a Nanodrop spectrophotometer (Thermo Scientific) and validated for quality with a Bioanalyzer (Agilent Technologies). It was then sequenced using the Illumina’s Hi-Seq 2000 platform.

### Assembly, annotation and differential expression analysis

After assessing the quality of the reads and removing low-quality reads, high-quality (Phred Score ≥20) paired-end and orphan reads were merged together and aligned to *B*. *rapa* genome using Tophat aligner [14]. The aligned and unaligned reads were separately subjected to *de novo* assembly using Trinity software (http://trinityrnaseq.sourceforge.net/) with default k mer size set to 25 bp [15]. To generate non-redundant full-length transcriptome (unigenes), the assembled transcripts with sequence length longer than 300 bp and >70% identity were clustered together using CD-HIT-EST v 4.6.1 clustering tool [16]. Further, high-quality reads were mapped back to the assembled transcriptome sequences for assembly validation. Reads were aligned using Bowtie with default parameters (http://bowtie-bio.sourceforge.net/index.shtml). Transcripts with ≤70% mapping coverage were discarded from the final assembly.

Annotation for all the unique transcripts was done using BLAST homology search against *B*. *rapa* CDS and protein sequences from BRAD (http://*Brassica*db.org/), *Arabidopsis thaliana* cDNA sequences (TAIR-10 database: ftp://ftp.*Arabidopsis*.org/home/tair/Sequences/blast_datasets/TAIR10_blastsets/) and the National Center for Biotechnology Information Non-Redundant (NCBI-NR) Protein database (http://pubmlst.org/cgi-bin/mlstanalyse/mlstanalyse.pl?site=pubmlst&page=nrdb). BLAST hits with e-value ≤0.001 and query coverage above 50% were considered as homologous proteins. A custom AWK (programming language) script was used for filtering reciprocal best hits. BLAST hits were processed to retrieve associated Gene Ontology (GO) terms describing biological processes, molecular functions, and cellular components using GO-Elite [17]. BGI Web Gene Ontology Annotation Plotting (WEGO) tool was used for plotting GO terms [18].

Expression levels of all the transcripts in the individual libraries (CTRL and SS) were assessed by mapping high-quality (HQ) filtered reads using BOWTIE2 [19]. Mapped reads were further normalized using DESeq method [20]. Rapa and Non-Rapa segregation was done based on the homology by performing BLAST of secondary assembly against *Brassica*_rapa_v1.2.pep and *Brassica*_rapa_v1.2.cds obtained from *Brassica* database (BRAD; http://*Brassica*db.org/). Normalization and differential expression analysis (≥2 folds and False Discovery Rate (FDR) ≤0.05) were carried out using DEseq [20].

### Functional categorization and pathway analysis

For functional categorization and pathway visualization, we used KEGG orthologs, identified using the KEGG Automated Annotation Server (KAAS), with default parameters (http://www.genome.jp/kegg/) and separately mapped the high-expressing (Normalized RPKM ≥ 100) and differentially expressed transcripts (FC ≥2, FDR ≤0.05) on the MapMan tool (http://mapman.gabipd.org/web/guest/mapman; [21]) with the Brapa_197 mapping file. Pathway images were saved as. png files.

### Quantitative real-time PCR (qPCR) validation

The gene-specific primers for qPCR were designed using Primer Express software (Applied Biosystems) and confirmed for their specificity by blast search in NCBI. For qPCR validation of differentially expressed unigenes, *B*. *juncea* var. CS52 seeds were germinated in vermiculite. Fifteen-day-old seedlings were subjected to 200 mM salinity stress and tissue was collected after 0 and 24 h of treatment. For comparative expression profiling, seeds of *B*. *juncea* var. CS52 and *B*. *nigra* were germinated in vermiculite. Tissue collected from fifteen-day-old seedlings was used for expression analysis. The RNA was isolated using TRIzol reagent (Sigma, USA) and treated with DNase as per manufacturer’s instructions. cDNA was synthesized using First Strand cDNA Synthesis Kit (Thermo Scientific). The data was normalized using *UBQ9* (Ubiquitin) as reference [22]. qPCR analysis was performed with two biological replicates. The relative expression values were calculated using 2^(-Delta Delta C(T))^ method [23]. The primer sequences used for validation purposes and comparative expression profiling have been given in S1 Table where S1A Table has list of primers used for qPCR validation of RNAseq data and S1B Table has list of primers used for comparative expression profiling of selected genes in *B*. *juncea* and *B*. *nigra* cultivars.

## Results

### RNA sequencing, assembly statistics and validation

**Fig 1:**
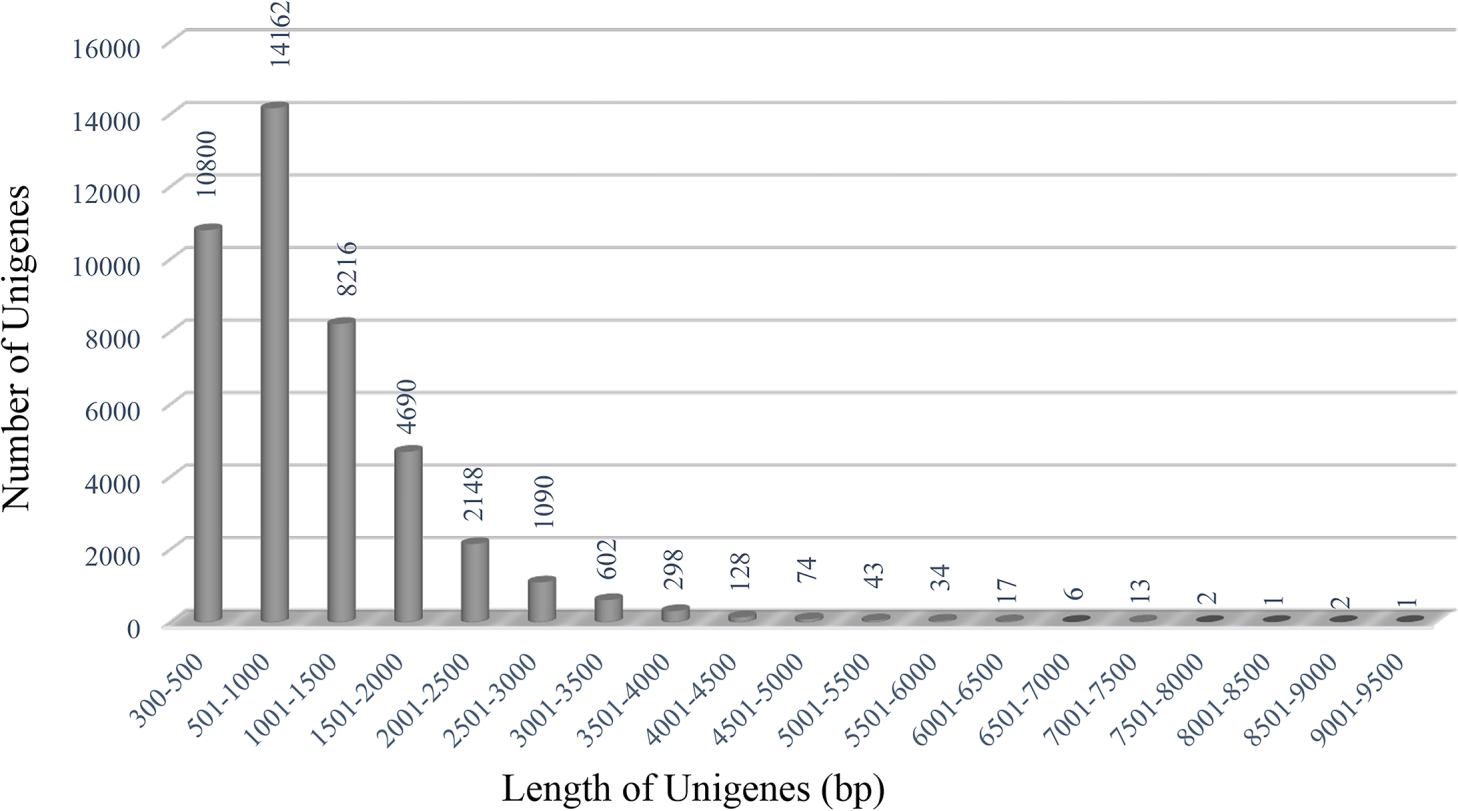
Sequence length distribution of unigenes predicted from assembled transcriptome of *B*. *juncea*. var. CS52.

We generated two cDNA libraries from the control (CTRL) and salinity stress-treated seedlings (SS) of *B*. *juncea* var. CS52. Transcriptome sequencing using Illumina paired-end sequencing platform yielded more than 200 million reads (103,827,526 & 98,442,584 from CTRL and SS libraries, respectively) of 100 bp (Table 1). After removing the low-quality reads, high-quality reads (94,800,630 from CTRL and 89,749,190 from SS library) were subjected to downstream analysis. We performed *de novo* assembly of the sequence data using Trinity assembler. The primary assembly resulted in 94,990 transcripts with N50 of 1042 bp. The N50 value improved to 1282 after running CD-HIT-EST with total number of transcripts reduced to 53,669 (Table 2). Based on the predicted transcripts, the GC content of *B*. *juncea* transcriptome is estimated to be 44.38% (Table 2). To validate these transcripts, we aligned reads from both the libraries to all the predicted transcripts. Transcripts with ≤70 mapping coverage were further discarded from the final assembly which yielded 47,513 transcripts, longer than 300 bp. Out of these, 42,327 transcripts with RPKM ≥1 were used for further analysis. Length distribution analysis of shortlisted 42,327 transcripts revealed 25.5% (10800) of them to be 300–500 bp in length, 33.4% (14162) are from 501 to 1000 bp, whereas, 41% (17365) are longer than 1000 bp (Fig 1).

**Table 1:** Summary of read statistics from RNA sequencing of *B*. *juncea* var. CS52.

	Total number of paired-end reads	Number of paired-end high quality reads (% total reads)	Number of Low quality reads (% total reads)
**CTRL**	103,827,526	94,800,630 (91.3%)	9,026,896 (8.7%)
**SS**	98,442,584	89,749,190 (91.2%)	8,693,394 (8.8%)
**Total**	202,270,110	184,549,820 (91.2%)	17,720,290 (8.8%)

**Table 2:** Statistics of the Trinity assembly after clustering.

Parameters	Values
Total number of transcripts	53,669
Transcriptome length (bp)	51,151,545
Minimum transcript length (bp)	301
Maximum transcript length (bp)	9029
Average transcript length (bp)	953
N50	1282
GC Percentage	44.38%

**Fig 2:**
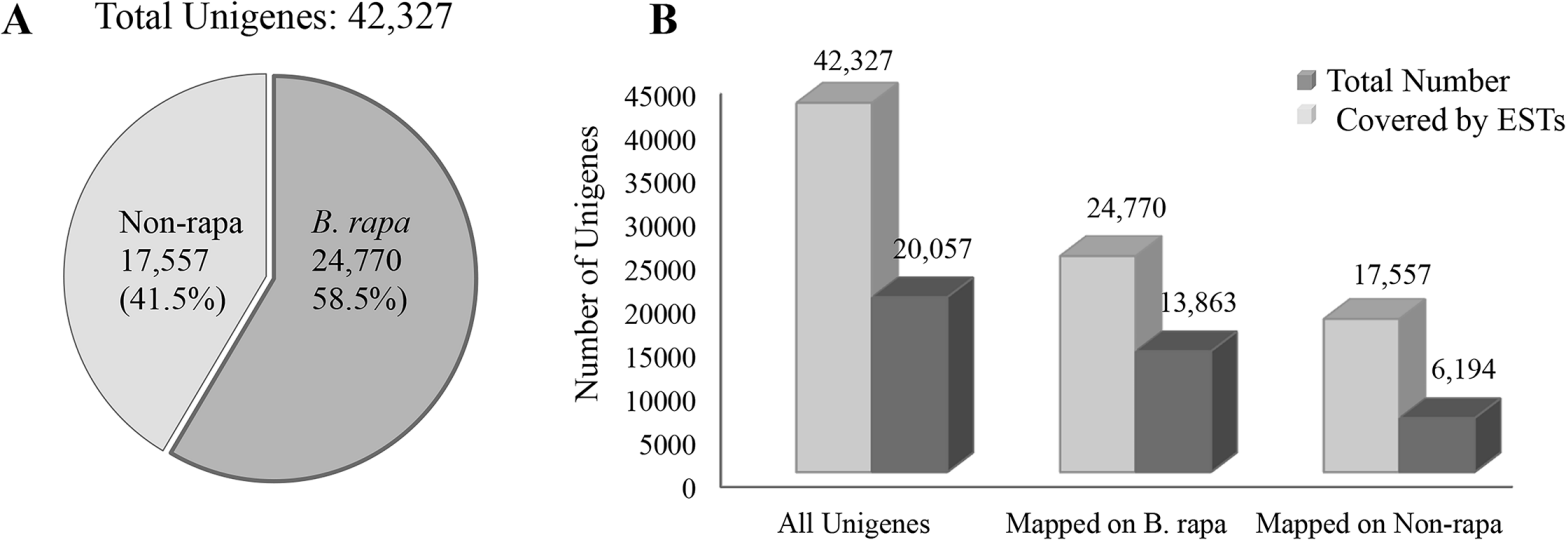
Mapping results of predicted unigenes on A) *B*. *rapa* transcripts and B) ESTs available for *B*. *rapa*, *B*. *nigra* and *B*. *juncea*. A) Pie chart showing proportion of total unigenes mapped on *B*. *rapa* transcripts and those which could not be mapped (non-rapa). B) Results of EST mapping on total unigenes, *B*. *rapa* mapped unigenes and non-rapa unigenes are presented in the form of bar graph.

Further, to determine the subgenome-specific transcripts, we mapped the 42,327 transcripts on *B*. *rapa* genome. About 58% (24,770) of these mapped on predicted transcripts of *B*. *rapa*, whereas, rest 17,557 (41.5%) likely originated from *B*. *nigra* and were categorized as non-rapa transcripts (Fig 2A). Further, similarity search analysis with *Arabidopsis* genome revealed homologous sequences for 48% (20,366) unigenes analyzed here.

To validate the assembly and gene predictions, we mapped all the EST sequences (221,810 in number) available for *B*. *rapa*, *B*. *nigra* and *B*. *juncea* in NCBI database onto the predicted transcripts. These included 214,482 *B*. *rapa*, 1810 *B*. *nigra* and 5518 *B*. *juncea* ESTs. Out of total 221,810 *Brassica* ESTs downloaded, 72.5% mapped on our assembled genome. With 20,057 (47.4%) of the 42,327 predicted transcripts validated by a corresponding EST, remaining 52.6% unigenes, identified in our study, are novel compared to previously available EST data (Fig 2B). Among those mapped to *B*. *rapa* genome, 56% could be validated by representative ESTs (Fig 2B). Only 35% of the non-rapa transcripts could be validated by representative ESTs due to very less number of EST information available for *B*. *nigra* (Fig 2B).

### Annotation and functional categorization of the predicted unigenes

**Fig 3:**
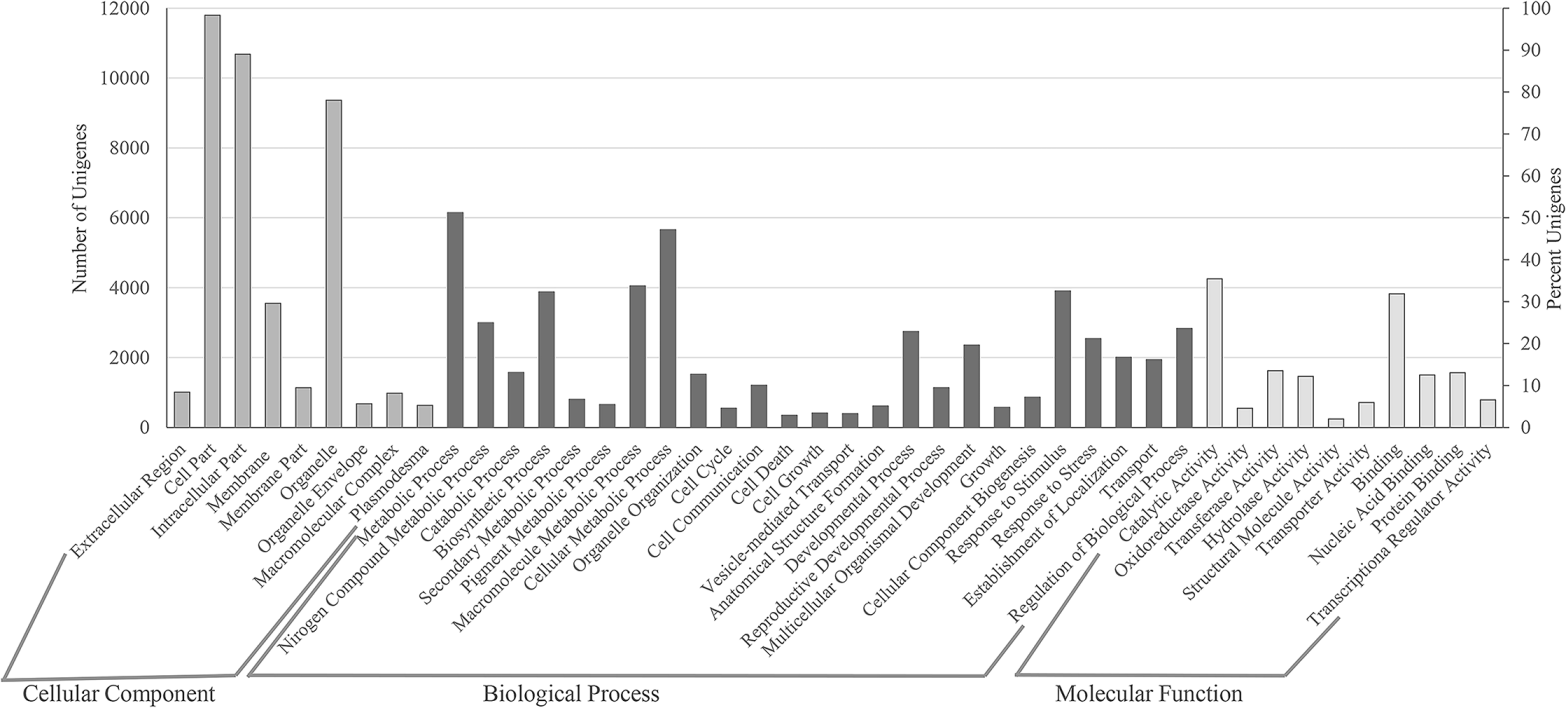
Histogram showing GOSlim terms assigned to *B*. *juncea* unigenes. The results are summarized for biological process, cellular component and molecular function categories. The left Y-axis represents the number of genes and the right Y-axis shows the percentage of total unigenes.

For annotating *B*. *juncea* unigenes, we performed sequence similarity search with Brassica Database (BRAD; http://*Brassica*db.org/brad/) that has sequence information for *B*. *rapa* genome followed by a blast search with *Arabidopsis* sequence data in The *Arabidopsis* Information Resource (TAIR; http://www.*Arabidopsis*.org/) and then Non-redundant database of proteins at NCBI (http://www.ncbi.nlm.nih.gov/protein). Out of 42,327 predicted unigenes, 58.2% (24,770) could be annotated by blast search with BRAD database with an E-value cut off of 1e^-3^ and minimum query coverage of 60%. Further, 2934 (7%) and 880 (2%) transcripts were annotated based on the similarity search in TAIR and NRDB databases, respectively. Combined together, 67% (28,584) transcripts could be successfully annotated. The functional classification of all the genes was carried out based on significant gene ontology terms (Fig 3). In the biological process category, the largest representation was of the metabolic processes followed by biosynthetic process and response to stimulus indicating that we have been able to capture stress-responsive transcripts in this study (Fig 3). In the molecular function category, catalytic activity was the largest group followed closely by binding activity (Fig 3).

### Changes in the transcript levels in response to salinity stress

Using the empirical criterion of ≥ two-fold change and FDR ≤0.05, a total of 1469 genes were differentially expressed in response to stress. Of these, 587 were up regulated, whereas, 882 were down regulated in response to stress (S2 Table). Majority of the genes (79% of up regulated genes and 75% of down regulated genes) exhibit differential expression ranging from 2 to 5 folds. Only 4% of up and 3% of down regulated genes exhibit more than 100 folds change in transcript accumulation in response to salinity stress. Most of the highly up regulated genes remain unknown except a basic helix loop helix transcription factor gene homologous to *Arabidopsis RITF1* (*RSA1 INTERACTING TRANSCRIPTION FACTOR 1*). RITF1 is a positive regulator of *SOS1* and several key genes involved in detoxification of reactive oxygen species generated in response to salt stress [24]. Notable among highly down regulated genes were two TRAF-like family genes and a LEA (Late Embryogenesis Abundant) gene, *LEA4-5*, which typically accumulates in response to water deficit conditions [25].

**Fig 4:**
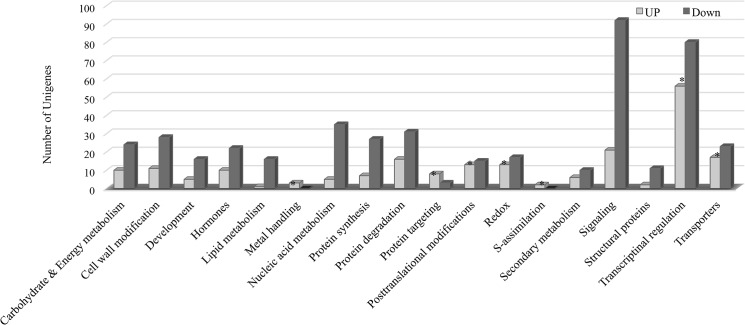
Pathway analysis showing relative significance of affected pathways for differentially expressed unigenes in response to salinity stress. The number of genes is represented on the Y-axis. The pathways enriched among up regulated genes are marked by *.

The differentially expressed genes were further analyzed for key pathways affected using the MapMan and KEGG databases. The genes involved in basic metabolic processes including carbohydrate & energy metabolism, plant development, lipid metabolism, nucleic acid metabolism, protein metabolism and signaling were down regulated (Fig 4). Whereas, those involved in metal handling, S-assimilation, protein targeting and posttranslational modification, transcriptional regulation and transporters were significantly up regulated (Fig 4).

**Fig 5:**
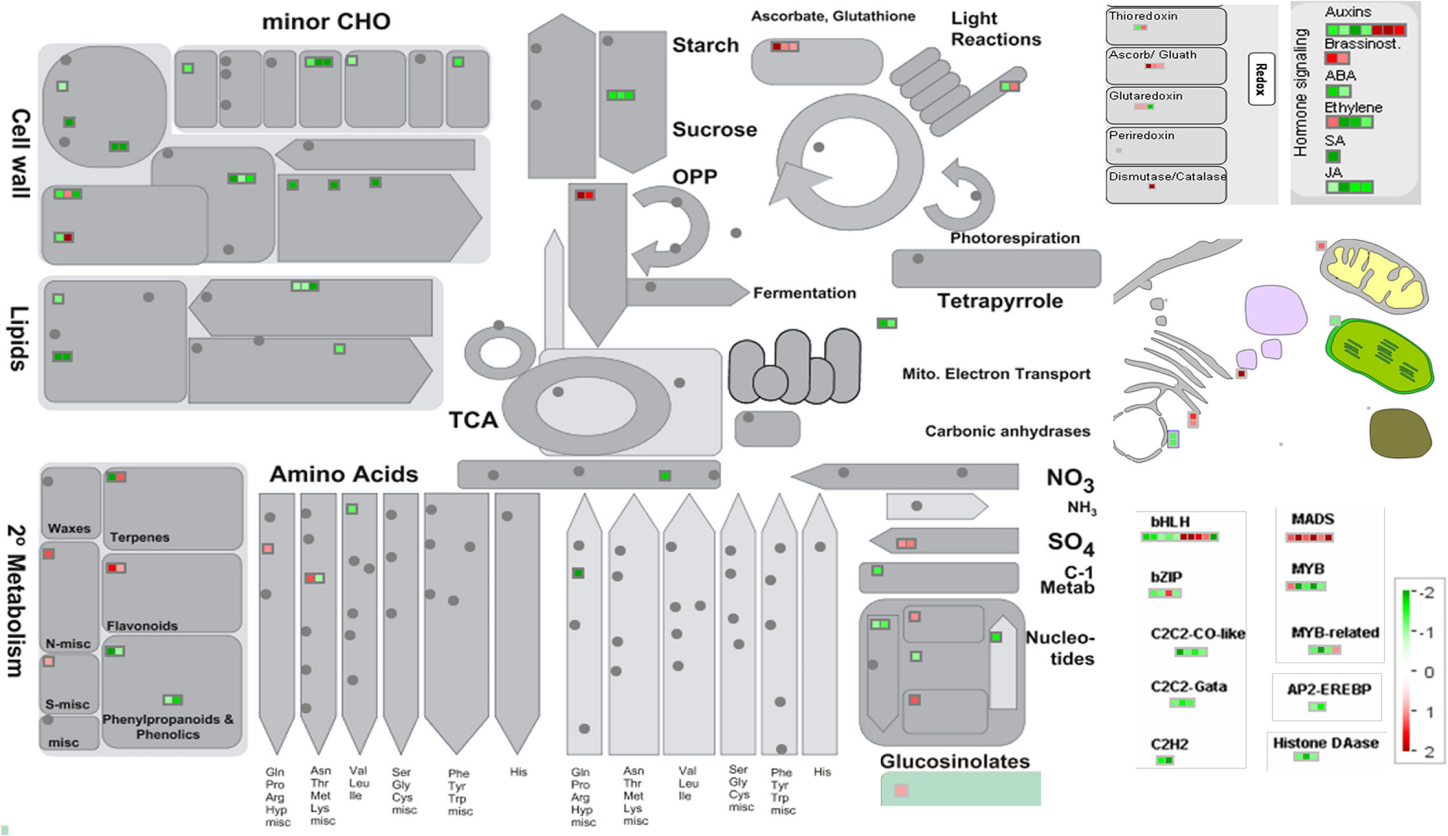
MapMan depiction of gene regulation in functional categories associated with different pathways. Distribution of unigenes into different pathways, generated in MapMan, are illustrated. Log_2_ fold changes are indicated by the color scale, red squares represent up regulated genes and green squares represent down regulated genes. The grey circles represent genes not differentially expressed in our study.

To further explore these pathways, we mapped differentially regulated genes on MapMan tool (Fig 5). Among the transcription factor family genes, zinc finger family genes, bHLH and MADS-box family transcriptions factors were particularly up regulated in response to salinity stress (Fig 5). Several genes mediating ROS detoxification including *superoxide dismutase* (*SOD*), *thioredoxin* (*Trx*), *dehydroascorbate reductase* (*DHAR*) and *glutathione peroxidase* (*GPX*) were also up regulated (Fig 5). Several key abiotic stress-responsive markers genes involved in sulfur assimilation (*ATPS* and *APR*), proline biosynthesis (*P5CS*), ABA biosynthesis (*NCED4*) and ion sequestration (*ENH1*) were also up regulated (Fig 5).

### Real-time qPCR-based validation of gene expression

**Fig 6:**
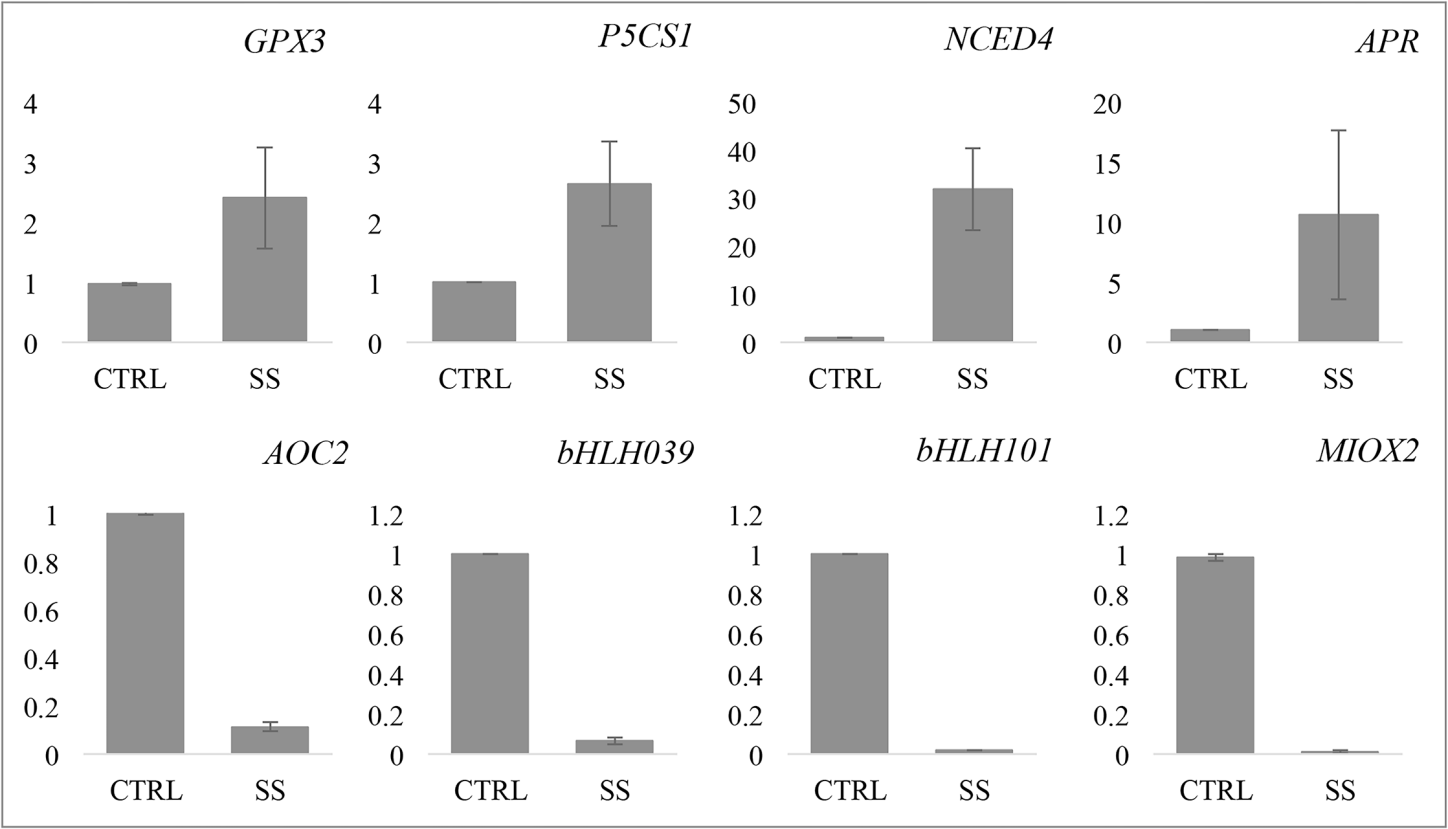
qRT-PCR validation of RNA-seq results. Expression of eight genes, showing varied expression patterns in RNA-sequencing experiment, were analyzed in CTRL as well as SS seedlings using qRT-PCR. The error bars show standard error between two biological replicates used for this experiment.

To validate the expression data obtained using transcriptome sequencing, we performed real-time qPCR with eight genes showing varied expression patterns (Fig 6). As reported, *UBQ9* exhibiting most stable expression in different developmental stages and treatments was used as reference for gene expression normalization [22]. All the four genes including *GPX3*, *P5CS1*, *NCED4* and *APR* upregulated in response to salt stress in transcriptome sequencing experiments showed significant up regulation in qPCR data also. Conversely, *allene oxide cyclase 2* (*AOC2*), *myo-inositol oxygenase 2* (*MIOX2*) and, bHLH transcription factor encoding genes, *bHLH039* and *bHLH101* showed down regulation in both transcriptome sequencing and qPCR experiments. Though the magnitude of fold change observed varied in different experiments, the overall regulation patterns obtained with qPCR were in agreement with the RNA-sequencing data (Fig 6).

### Identification and analysis of most abundantly expressed transcripts under normal growth conditions

**Fig 7:**
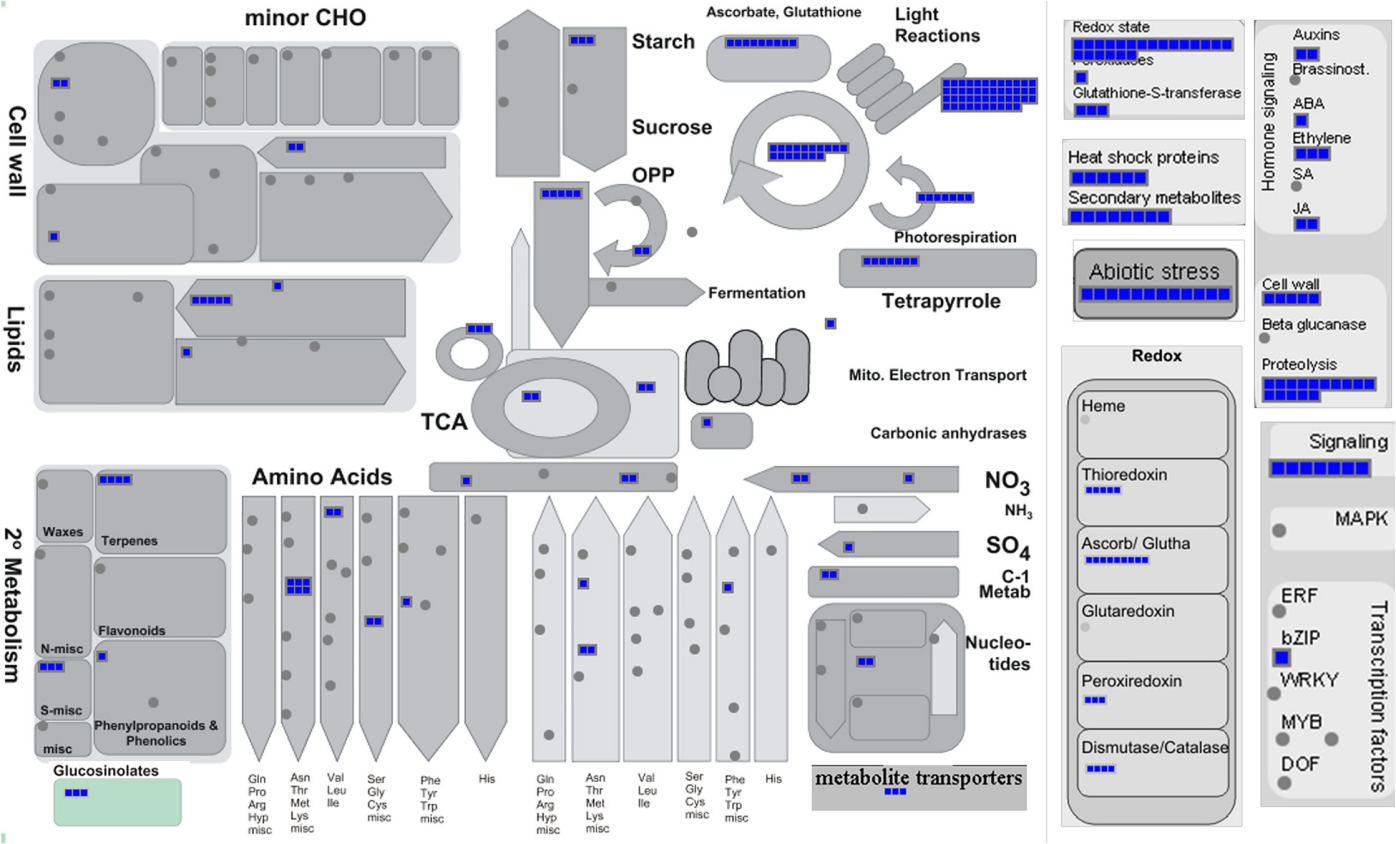
Mapping of high expressed unigenes on MapMan pathways. The genes exhibiting high expression (≥100 normalized RPKM values) were mapped on functional bins assigned to different pathways in MapMan. Blue squares represent high expressed genes. Grey circles represent genes not exhibiting high expression (≥100 normalized RPKM) in our dataset.

**Fig 8:**
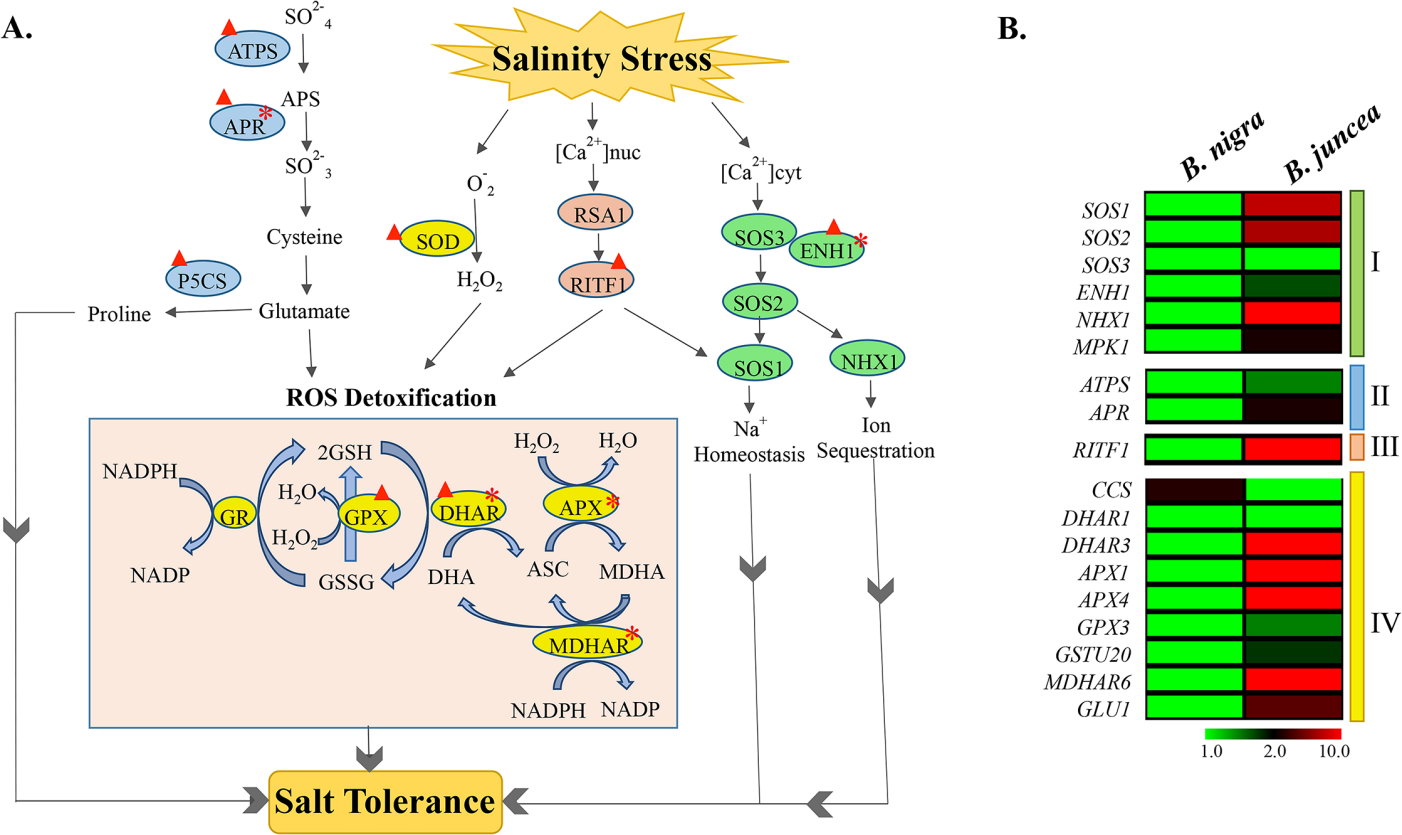
Schematic illustration of various cellular pathways mitigating salt stress in *B*. *juncea* var. CS52. A) Schematic overview of key pathways and genes affected in response to salinity stress in *B*. *juncea* is presented. Information about highlighted genes was gathered from published articles. Up regulated genes and those exhibiting high expression in absence of stress are highlighted with a red upward arrow and star, respectively. ASC: Ascorbate; GSH: Glutathione; ATPS: ATP sulfurylase; APR: Adenosine 5'-phosphosulfate reductase; P5CS: Delta1-pyrroline-5-carboxylate synthetase; SOD: Superoxide dismutase; GR: Glutathione reductase; GPX: Glutathione peroxidase; DHAR: Dehydroascorbate reductase; APX: Ascorbate peroxidase; MDHAR6: Monodehydroascorbate reductase 6; AtGSTU20: Glutathione S-transferase TAU 20; GLU1: Glutamate synthase 1; CCS: Copper chaperone for superoxide dismutase; RSA1: Short Root In Salt Medium 1; RITF1: RSA1 Interacting Transcription Factor 1; SOS3: Salt Overly Sensitive 3; SOS2: Salt Overly Sensitive 2; SOS1: Salt Overly Sensitive 1; ENH1: Enhancer of sos3-1; NHX1: Sodium/Hydrogen exchanger 1; MAPK1: Mitogen-activated protein kinase. B) Heat map showing comparative expression patterns of candidate genes in *B*. *juncea* and *B*. *nigra* seedlings under normal growth conditions. Log_2_ expression values are indicated by the color scale with red representing high expression, black representing medium expression and green representing low level expression. The colored bars on the right demarcate genes representing different pathways. I: Ion homeostasis and sequestration; II: Sulfate assimilation; III: Gene Regulation and IV: ROS detoxification.

For the unigenes predicted in this study, RPKM values ranged from 0.009 to 50,474 for CTRL and 0.01 to 63,215 for SS library, respectively indicating dynamic range of expression. Analysis of expression dynamics in CTRL seedlings revealed about 80% unigenes had normalized RPKM of less than 10. About 17% transcripts express at medium levels with an RPKM ranging from 10 to 50 and 2% exhibit high expression with RPKM from 50 to 100. Only 1.7% of total unigenes had RPKM values greater than 100 (S3 Table). To determine the key pathways exhibiting high expression in a salt-tolerant variety under normal growth conditions, we mapped top 733 highly-expressed unigenes (RPKM ≥100) on functional pathways using MapMan tool (Fig 7). Although, as expected, a major portion of these were photosynthetic genes and those regulating basal metabolic functions, several genes involved in nitrate assimilation, sulfur assimilation and glutathione metabolism are also highly expressed (Fig 7). Notable of these are *catalase*, *superoxide dismutase*, *ascorbate peroxidase* (APX), *monodehydroascorbate reductase* (*MDHAR*), *DHAR* and *glutamate synthase* (*GLU1*) (Figs 7 and 8A). Most of the high expressed transcripts show little variation in both the libraries. But, nine of the high-expressed transcripts in CTRL library exhibit more than two fold induction in response to stress. These include *enhancer of sos3-1* (*ENH1*), *APS reductase 1* and a GA-responsive GAST1 protein homolog. Also, 17 genes exhibiting high expression in CTRL samples were down regulated by >2 folds in response to stress. Notable of these were an f-type thioredoxin, a xyloglucanendotransglucosylate/hydrolase, a fasciclin-like arabinogalactan-protein 9 (*Fla9*), two lipid transfer proteins and homolog of *Arabidopsis HARMLESS TO OZONE LAYER 1* (*HOL1*) that is involved in glucosinolate metabolism and disease resistance [26].

### Comparative expression profiling of candidate genes in *B*. *juncea* and *B*. *nigra*

To identify the key genes regulating salinity tolerance, we performed a comparative expression analysis of eighteen abiotic stress-responsive genes in two contrasting genotypes of *Brassica* (Fig 8B). Earlier, we had reported morphological, physiological and biochemical analysis of eight genotypes of *Brassica* in response to 24 h of salinity stress [27]. Our results showed sharp contrast in overall growth, electrolyte leakage, proline accumulation, K^+^/Na^+^ ratio and transcriptional pattern of SOS members in salt-sensitive diploid species *B*. *nigra* and salt-tolerant *B*. *juncea* var. CS52 [27]. Therefore, we used these two genotypes to analyze the comparative expression profile of candidate genes in absence of stress using qPCR. The tissue was sampled from fifteen-day-old seedlings grown under normal growth conditions from both the genotypes. Our results revealed that most of the redox and abiotic stress responsive-genes except copper chaperone of superoxide dismutase (*CCS*) express at high levels in seedlings of salt-tolerant genotypes of *B*. *juncea* compared to salt-sensitive *B*. *nigra* in the absence of stress (Fig 8B). These results indicate constitutive expression of abiotic stress responsive genes in *B*. *juncea* var. CS52.

## Discussion

In absence of whole genome sequence, RNA sequencing is a very successful application of next generation sequencing technologies for annotating the whole genome and quantitative analysis of gene expression [28]. In this study, we implemented RNA sequencing technology to get insights into the stress transcriptome of a salt-tolerant variety CS52 of *B*. *juncea* at seedling stage. Salinity tolerance at seedling stage is particularly important as most of the crop plants are most sensitive to environmental stresses at seedling and flowering stages. Earlier, we had analyzed several physio-morphological, biochemical and molecular aspects of salinity tolerance in eleven cultivars of *Brassica* at seedling stage [27]. Our results showed least penalty in terms of growth reduction and, electrolyte leakage with the highest accumulation of proline and favorable maintenance of K^+^/Na^+^ ratio in *B*. *juncea* seedlings compared to other cultivars analyzed [27]. Also, a positive correlation between expression pattern of salt overly sensitive members and stress tolerance was reported in *Brassica* genotypes in response to 24 h of 200 mM salinity stress [27]. Therefore, to elucidate the molecular aspects of salinity tolerance and investigate the relationship between physiological response and transcriptional dynamics, we generated RNA-seq libraries from CS52 variety of *B*. *juncea* seedlings under normal growth conditions and in response to 24 h of salinity stress. We obtained over 200 million reads leading to 53,669 predicted unigenes. To eliminate potential false positives reads due to sequencing errors, statistical mapping error or trace amount of genomic DNA contamination, only 42,327 high-confidence unigenes, longer than 300 bp with RPKM >1, were used for downstream analysis. Only 47.4% of these are represented by *Brassica* ESTs in public databases indicating that 52.6% of the unigenes, predicted in this study, are novel compared to EST sequence available for *B*. *juncea* and constituent genomes (*B*. *rapa* and *B*. *nigra*).

Since whole genome of *B*. *rapa* has already been sequenced and *Brassica* species share very close genetic relationship with *Arabidopsis*, we took advantage of the annotation information available for *B*. *rapa* and *Arabidopsis* for annotation of the predicted genes. Using these resources and protein database at NCBI, we could annotate 67% of the predicted unigenes. The annotation of rest 33% unigenes remains unknown. Exploring this unknown territory remains a challenge for the whole plant community as significant insights might be residing in this part of the transcriptome.

Further, differential expression analysis revealed significant up regulation of several pathways including sulfur assimilation, proline accumulation, ROS detoxification and calcium signaling associated with salinity stress response. The expression of ABA biosynthetic enzyme coding gene, *9-cis-EPOXYCAROTENOID DIOXYGENASE 4* (*NCED4*), was elevated in response to salinity stress, whereas, the genes involved in photosynthesis, carbohydrate metabolism and jasmonic acid biosynthesis were mainly down regulated likely as an adaptive response to divert resources to combat with abiotic stress and prevent photodamage.

Sulfur assimilation involves activation of sulfate to adenosine 5´-phosphosulfate (APS) by ATP sulfurylase (ATPS). Subsequently, APS is reduced to sulfite by APS reductase (APR). Sulfite is reduced to sulfide and incorporated into cysteine which is direct precursor for the synthesis of glutathione (GSH). We observed significant increase in the transcript levels of both *ATPS* and *APR* genes in response to salinity stress (Fig 8). In fact, APR is among the highly expressed genes in *B*. *juncea* seedlings even in the absence of stress with expression of both genes much higher in tolerant cultivar compared to sensitive one (Fig 8).

GSH acts as a sensor of redox and maintain lower levels of ROS *via* the glutathione-ascorbate cycle [29,30]. The glutathione-ascorbate cycle involves several antioxidant metabolites and enzymes. Superoxide dismutase (SOD) catalyze dismutation of toxic superoxide radical into oxygen or hydrogen peroxide (H_2_O_2_). Oxidation of ascorbate (ASC) to monoaldehydroascorbate (MDHA) by ascorbate peroxidase (APX) scavenges H_2_O_2_. MDHA is either directly converted back to ASC by monodehydroascorbate reductase (MDHAR) or converted to dehydroascorbate (DHA) which is again converted to ASC by dehydroascorbate reductase (DHAR). DHAR uses GSH which is regenerated from its oxidized form GSSG by action of glutathione reductase (GR), glutathione peroxidase (GPX) or glutathione S-transferase (GST), leading to removal of ROS. We observed significant induction of several ROS detoxification genes including *SOD*, *GPX*, *DHAR*, *APX*, *MDHAR* in response to stress though their initial transcript levels in *B*. *juncea* were also much higher compared to those in salt-sensitive cultivar (Fig 8). We also observed up regulation of *delta1-pyrroline-5-carboxylate synthetase* (*P5CS*) gene that catalyzes the rate-limiting step in proline biosynthesis in response to salinity stress [31,32]. These observations are in agreement with the previous reports suggesting constitutive expression of abiotic stress responsive genes in tolerant varieties as a major factor contributing to stress tolerance [8,32,33].

Earlier studies proposed that efficient Salt Overly Sensitive (SOS) pathway that comprises *SOS3*, *SOS2* and *SOS1* could be a major factor in determining salt tolerance in *Brassica* genotypes [7,27]. SOS3, a calcium binding protein, senses increase in cytosolic calcium and interacts with SOS2 protein kinase. The SOS2-SOS3 protein complex then phosphorylates and activates a plasma membrane localized Na+/H+ antiporter SOS1 [34]. In agreement with the previous studies, we also observed higher expression of SOS genes in *B*. *juncea* compared to *B*. *nigra* seedlings under normal growth conditions (Fig 8). However, we did not observe significant change in their transcript levels in response to salinity stress. As proposed earlier, the reason for not much induction of SOS genes in response to stress could be the fact that SOS genes are mostly regulated at posttranslational level [35]. Notably, enhancer of SOS3 pathway, *ENH1*, exhibits very high transcript levels in *B*. *juncea* in the absence of stress and is further up regulated in response to salinity stress.

Recently, a nuclear calcium sensing pathway comprising of a calcium binding protein, RSA1 (SHORT ROOT IN SALT MEDIUM 1) and a bHLH transcription factor, RITF1 has been shown to play critical role in stress tolerance in *Arabidopsis* [24]. The RSA1-RITF1 complex regulates expression of key genes involved in detoxification of ROS and maintaining Na^+^ homeostasis in response to salt stress [24]. *RITF1* was among the top 1% genes exhibiting 128 folds up regulation in response to stress in this study suggesting that it plays very important role in regulating gene expression in response to salinity stress and may serve as a molecular marker of stress tolerance. *RITF1* also regulates the expression of *SOS1* indicating a link in both the calcium signaling pathways. Ultimately, activation of antioxygenic enzymes seems to be the major reason for improved growth under salinity stress condition by preventing oxidative damage. Also, since oxidative stress is a secondary effect of several biotic and abiotic stress factors, it would be interesting to investigate if CS52 variety of *B*. *juncea* is resistant to other stresses as well.

In conclusion, our results show that multiple pathways may be involved in salinity tolerance in *B juncea* var. CS52. The data generated in this study will serve as a valuable resource to support genome analysis, develop expression arrays, molecular marker identification and, initiating functional and comparative genomic studies in *B*. *juncea*.

## Supporting Information

S1 Table List of primers used for quantitative PCR. (XLSX)

S2 Table List of up- and down-regulated transcripts. (XLSX)

S3 Table List of transcripts with normalized RPKM ≥100. (XLSX)
